# Safety and feasibility of paired vagus nerve stimulation with rehabilitation for improving upper extremity function in people with cervical spinal cord injury: study protocol for a pilot randomized controlled trial

**DOI:** 10.3389/fneur.2024.1465764

**Published:** 2024-11-14

**Authors:** Nuray Yozbatiran, Gerard E. Francisco, Radha Korupolu

**Affiliations:** ^1^Neuromodulation and Neural Interfaces Laboratory, UTHealth NeuroRecovery Research Center at TIRR Memorial Hermann, Houston, TX, United States; ^2^Department of Physical Medicine and Rehabilitation, McGovern Medical School, The University of Texas Health Science Center at Houston, Houston, TX, United States; ^3^TIRR Memorial Hermann Hospital, Houston, TX, United States

**Keywords:** vagus nerve stimulation, spinal cord injury, rehabilitation, upper extremity, motor recovery

## Abstract

**Introduction:**

Pairing vagus nerve stimulation with traditional rehabilitation therapies results in improved motor recovery in people with stroke. However, this approach has not yet been studied in people with spinal cord injury (SCI). Motor recovery continues to be challenging after SCI, and there is a need for innovative research strategies to enhance motor recovery after SCI. Hence, this pilot randomized controlled trial aims to evaluate the safety, feasibility, and potential efficacy of pairing vagus nerve stimulation (VNS) with rehabilitation therapy to restore the motor function of the paretic upper limbs in people with cervical SCI.

**Methods and analysis:**

In this triple-blind, randomized, sham-controlled pilot study, 8 adults with chronic incomplete SCI will be implanted with a VNS device and randomly assigned to either active VNS (0.8 mA) control VNS (0.0 mA) paired with upper limb rehabilitation. Each participant will undergo 18 in-clinic therapy sessions over 6 weeks, each lasting 120 min and delivered three times per week. Following the in-clinic phase, participants will continue with a 90-day home exercise program. Participants in both groups will receive similar goal-directed and intense upper limb rehabilitation. The therapy is focused on active movements, task specificity, high number repetitions, variable practice, and active participant engagement. Post-treatment assessment will occur immediately after in-clinic therapy and at 30 and 90 days of follow-up. After completion of blinding at 90 days follow-up, participants in the control group will be offered 6 weeks of in-clinic active VNS (0.8 mA) paired with rehabilitation. The safety of pairing VNS with rehabilitation will be assessed by the occurrence of adverse events in each group, and feasibility by the number of treatment sessions and follow-up visits attended and the number of dropouts. Potential efficacy will be assessed by measuring the change in Graded Redefined Assessment of Strength, Sensibility and Prehension (GRASSP) performance from baseline to immediately after in-clinic therapy and to 90 days. Secondary clinical outcome measures are the Toronto Rehabilitation Institute Hand Function Test, Capabilities of Upper Extremity Questionnaire, Spinal Cord Injury Independence Measure-III self-care subscore, and Spinal Cord Injury-Quality of Life scale.

**Ethics and dissemination:**

The trial protocol was approved by the Institutional Review Board of UTHealth (HSC-MS-22-0579). We anticipate publishing the results in a peer-reviewed journal within 1 year of study completion.

**Clinical trial registration:**

ClinicalTrials.gov, NCT05601661.

## Introduction

Worldwide, an estimated 768,473 persons (95% CI 597,213–939,732) sustain a traumatic spinal cord injury (SCI) each year (1). Data from the United States show that complete or incomplete cervical SCI (also called complete or incomplete tetraplegia) accounts for more than 50% of new SCI cases (2). Cervical SCI, which results in paralysis of all four extremities, greatly limits a person’s ability to live independently and perform basic activities of daily living, like eating, bathing, dressing, using the toilet, and transferring in and out of bed or a chair (3, 4). Consequently, people with cervical SCI with severe impairments in their upper extremity motor function require significant assistance and caregiver support, resulting in enormous lifetime direct and indirect costs (5–7). Individuals with cervical SCI report regaining arm and hand function as one of their top priorities for improving their quality of life (8).

Rehabilitation is the most common approach to restoring motor function, and current therapeutic interventions for restoring upper extremity motor function after SCI are limited. Recent evidence suggests that most recovery after SCI is a result of synaptic plasticity in spared neural pathways above and below the level of the SCI (9–11). *Neuroplasticity* is the ability of spared neural cells and pathways to change in response to intrinsic and extrinsic factors and is a key mechanism for functional recovery after neurological injury (12, 13). In the last decade, interest has grown in pairing rehabilitation therapy with various neuromodulation interventions such as vagus nerve stimulation (VNS) to optimize neuroplasticity and functional recovery.

Vagus nerve is one of the important cranial nerves that carries parasympathetic and branchial motor efferents to several target organs. However, a large portion of the vagus nerve consists of afferent connections to several nuclei in the brain stem and is known to foster a neurochemical milieu that facilitates release of neuromodulators, including acetylcholine, norepinephrine, serotonin, and brain-derived neurotrophic factors which promote cortical plasticity. The repeated pairing of brief bursts of VNS with sensory or motor events induces large-scale expansion of cortical representations compared to interventions without VNS pairing (14–19). These effects of VNS paired with rehabilitation have been extensively studied in animal models of stroke, traumatic brain injury, and SCI (20–28). These studies consistently showed that pairing VNS with rehabilitation improves cortical plasticity, and upper extremity recovery was enhanced compared with identical rehabilitation training without VNS. Preclinical studies on the dosing and timing of VNS suggest that delivery of rapid bursts of VNS immediately after a motor event using 0.8 mA at 30 Hz frequency is required to maximize the cortical plasticity and recovery of upper extremity motor function (21, 24, 27, 28).

A recent large multisite randomized controlled trial (RCT) of paired VNS with rehabilitation compared with sham VNS with rehabilitation in persons with stroke revealed greater improvement in hand function in the paired VNS group (29). Furthermore, the participants were able to successfully use VNS in the home setting during exercise, resulting in further improvement in upper extremity hand function as measured by Fugl-Meyer assessment. There are no data from human trials in persons with SCI. However, results of preclinical SCI animal studies suggest similar benefits of paired VNS and rehabilitation in incomplete cervical SCI, including animal models with injury to alpha motor neurons of distal limb musculature (26, 27). In another SCI animal study, effects of VNS on blood pressure and heart rate revealed no autonomic dysreflexia events, which is a known complication in response to noxious stimuli in people with cervical SCI. Additionally, although there was no reduction in blood pressure, a transient decrease in heart rate was noted, which immediately returned to baseline after VNS was stopped (27). Additionally, VNS has been shown to decrease symptoms of depression and pain and reduce inflammation in non-SCI populations, which are known complications of SCI (4, 30–35).

Therefore, we propose conducting a pilot study to evaluate the safety and feasibility of pairing VNS with rehabilitation therapy to restore the motor function of the paretic upper limbs in people with cervical SCI. A second outcome is to determine the potential efficacy of pairing VNS with rehabilitation as shown by measures of upper extremity strength, dexterity, and functional abilities. Additionally, we will explore effects on pain and depression. Our goal is to refine the protocol to address any challenges and barriers encountered during this study in a SCI population for a larger RCT testing the effectiveness of paired VNS therapy.

## Methods and analysis

The study protocol has been developed according to the SPIRIT 2013 reporting guidelines for clinical trials (36).

### Trial design

**Figure 1:**
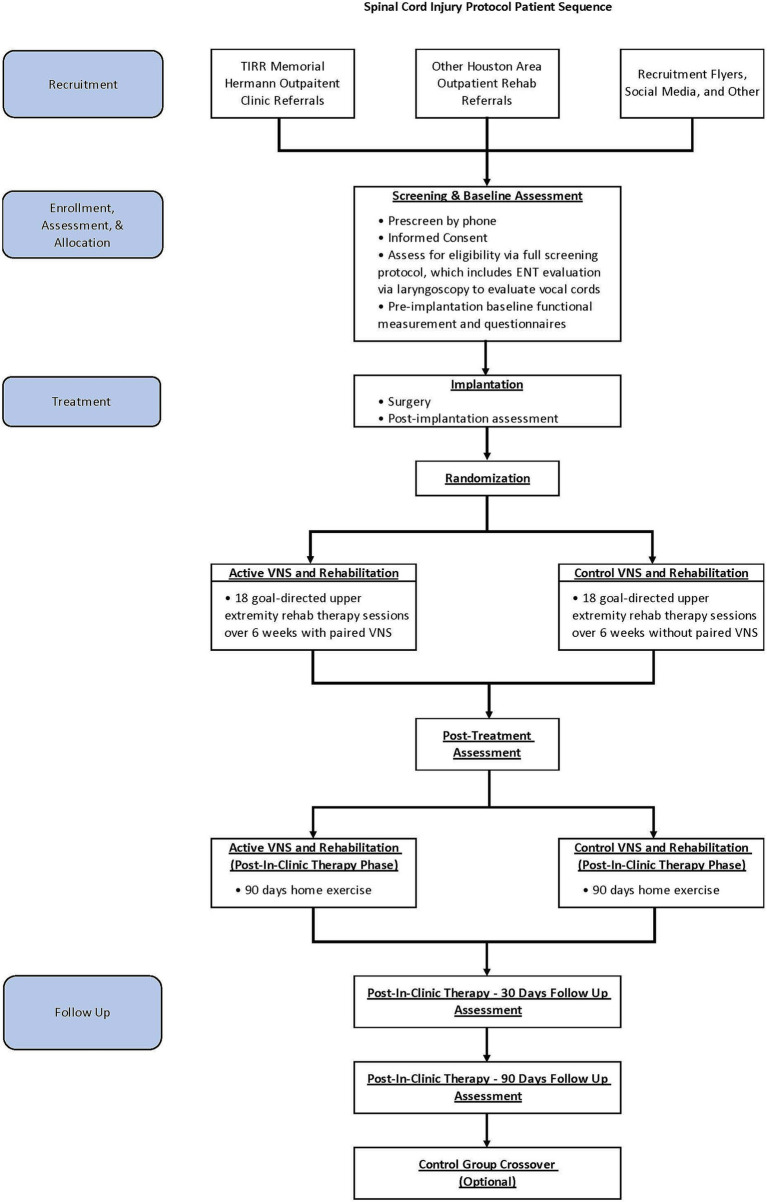
Study flow.

In this single-site, triple-blind, randomized, sham-controlled pilot study, adults with chronic incomplete SCI will be implanted with a VNS device and randomly assigned in a 1:1 allocation ratio to either active VNS + rehabilitation or control VNS + rehabilitation (Figure 1). Participants, treating therapists, outcome assessors, and investigators will be masked to group assignment. The methods and procedures will be similar to those in the pivotal study in stroke rehabilitation except for the outcome measures (29, 37). In this study, we will use SCI-specific validated measures.

The study is currently taking place at the NeuroRecovery Research Center at TIRR Memorial Hermann. Participants will be enrolled after screening and upon consent between 1 June 2023 and 30 December 2024. We anticipate that all study-related activities, including follow-up assessment, will be completed by 1 June 2025.

For the intervention, participants in both groups will receive 18 goal-directed upper extremity rehabilitation therapy sessions with or without paired VNS over 6 weeks, which will be followed by a 90-day home exercise program. Post-treatment assessment will occur immediately after in-clinic therapy (post-day 1, primary endpoint analysis time point) and will be repeated at post-30 days and post-90 days. After the post-90-day time point, participants in the control group will be offered 6 weeks of in-clinic active VNS paired with rehabilitation. We will obtain baseline and immediate post-therapy assessments for crossover control participants.

### Study population

Participants will be recruited locally through TIRR Memorial Hermann’s outpatient clinics and through outpatient rehabilitation facilities throughout the Houston metropolitan area.

To be eligible for the study, individuals must meet *all* the following inclusion criteria: (1) have a diagnosis of traumatic incomplete (American Spinal Injury Association Impairment Scale B-D) cervical SCI (C8 and above), (2) be at least 12 months post-traumatic SCI and within 5 years, (3) adults 18 years of age or older, (4) demonstrate some residual movement in the upper limb (e.g., able to perform pinch movement with thumb and index finger with or without tenodesis), and (5) meet all clinical criteria for surgical VNS implantation as determined by the principal investigator (PI), neurosurgeon, and anesthesiologist. Several confounding factors during the acute stage of SCI, such as spontaneous recovery and the high incidence of SCI-related complications, exist. To recruit a relatively homogenous population in this smaller trial, we kept the inclusion criteria 1–5 years post-SCI. As we gain knowledge of the safety and effects of VNS, we will reassess the inclusion criteria in a future trial.

Participants will be excluded if they (1) have nontraumatic SCI, (2) have ongoing dysphagia or swallowing difficulties, (3) show evidence of pre-existing vocal cord paralysis as determined by a laryngoscopy, (4) have a history of prior left-sided anterior cervical surgery with too much scar tissue at the surgery site as determined by the neurosurgeon, (5) have concomitant clinically significant brain injury, (6) have a history of prior injury to a vagus nerve, (7) are receiving medication that may significantly interfere with the actions of VNS on neurotransmitter systems at study entry (a list of excluded medications is available), (8) have other comorbidities or complications that will hinder or contraindicate the surgical procedure, (9) have medical or mental instability, or (10) are pregnant or plan to become pregnant during the study period. These criteria were selected to ensure that motor impairments of patients do not interfere with the ability to perform proposed functional tests.

### Screening and baseline assessment

Potential candidates will be screened by RK and NY and the research coordinator. Once study eligibility is determined based on preliminary inclusion criteria, then informed consent will be obtained. After the consent process, each participant will undergo further screening, which includes laryngoscopy evaluation of the vocal cords and review of medical history and physical examination by various clinicians on the study team to determine the safety of VNS implantation. Once eligible participants meet all study-related inclusion and exclusion criteria, pre-and post-implantation baseline assessments will be performed. The latter will be used as the main comparison point for statistical analysis.

#### Randomization and blinding

Participants will be randomized at implant surgery to either paired activeVNS and rehabilitation or paired control VNS and rehabilitation groups in a 1:1 ratio. Randomization will be performed in the REDCcap (Research Electronic Data Capture) by the research coordinator (38, 39). Participants, outcome assessors, statistician, and principal investigators will be blinded to the group assignment.

### Description of the study device

**Figure 2:**
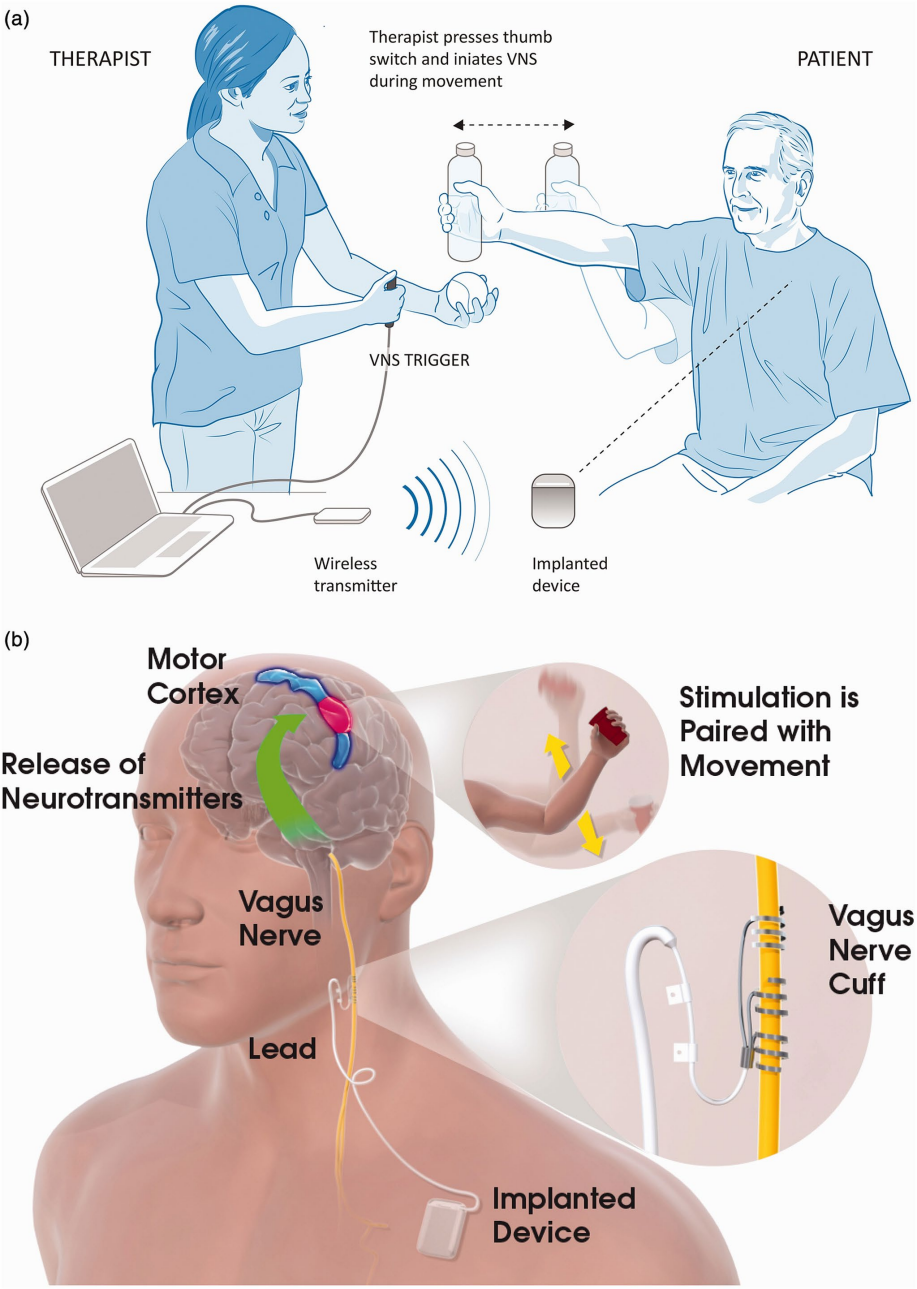
Vagus nerve stimulation (VNS) with rehabilitation. (a) In-clinic rehabilitation session set-up. The therapist is shown holding the VNS trigger button, which delivers a VNS pulse when the participant performs the task-specific movements. Also depicted in the figure is the wireless transmitter on the table connected to the notebook via a USB cable. When the therapist presses the VNS trigger button, the wireless transmitter sends a signal to the implanted device, which stimulates the left vagus nerve via a cuff electrode in the participant’s neck. (b) Possible mechanism for VNS paired with movement. Stimulation of the vagus nerve stimulates the deep brain cholinergic nucleus basalis and noradrenergic locus coeruleus neurons (base of green arrow). Stimulation of the vagus nerve during task-specific movements modulates the activity of the motor cortex (blue and red area) in a task-specific manner. Reprinted with permission from Kimberley et al. (37).

We will use an implantable system consisting of a neurostimulator [model 1001 implantable pulse generator (IPG)] and an implantable lead and electrode (model 30000 VNS lead). An external system consisting of a controller (model 2000 wireless transmitter) and software system (computer and model 4001 MicroTransponder SAPS software) will provide clinical control of settings for the IPG (Figure 2). All VNS devices (Vivistim System) will be donated by MicroTransponder Inc. (Austin, TX). The device and implant procedure proposed in this study (Vivistim System) is similar to systems approved by the Food and Drug Administration (FDA) for epilepsy and major chronic depression and is exactly the same as recently approved by the FDA for people with stroke for improving upper extremity function (40). All surgical procedures will occur at Memorial Hermann Hospital, Houston, Texas, and be performed by an experienced neurosurgeon and his clinical team.

### Device implant surgery

An experienced neurosurgeon will perform device implantation. Expectations are that general anesthesia will be used. Electrodes will be implanted on the left cervical portion of the vagus nerve. The IPG will be implanted in a subcutaneous pocket below the left clavicle.

The lead connector will be tunneled between the cervical and infraclavicular incisions, and the electrodes attached to the nerve. The lead is looped in a gentle curve and sutured through a silicone retainer adjacent to soft tissue to avoid tension on the lead. A second loop is made superficially and sutured to the sternocleidomastoid fascia. The distal terminals of the tunneled bipolar leads are connected to the IPG. The system is then tested to confirm a good electrical connection, and the IPG is placed in its pocket with excess lead coils positioned posteriorly to minimize the possibility of damage if the incision is reopened for device replacement.

The recovery period is expected to be from 1 to 24 h, and participants will return home the same day. Participants will be released after medical clearance only if there is a person to drive them home. Participants will recover for approximately 3–7 days before testing begins, depending on the investigator’s medical opinion and scheduling.

### Intervention

Participants in the active VNS group will receive one 0.5-s stimulus of VNS during upper extremity task training throughout the treatment session (120 min). Those in the control group will receive VNS only at the start of each session (for the first 4 movements). In-clinic rehabilitation will occur 3 days a week for 6 weeks, for 18 sessions. All sessions will be performed at the NeuroRecovery Research Center at TIRR Memorial Hermann, Neuromodulation and Neural Interfaces Lab. The stimulation output current is set to 0.8 mA, with 100 μs pulse width and a frequency of 30 Hz. Stimulation parameters were chosen based on VNS studies in humans with stroke, and these parameters were tolerable without any side effects (29). The device is programmed to stimulate for 0.5 s on each button push. Participants in each treatment group (active vs. sham stimulation) will receive 5 stimulations in reducing strength (starting at 0.8 mA and then reducing to 0.1 mA each step) at the beginning of each therapy session, followed by a paired stimulation according to randomized allocation (0.8 mA vs. 0.0 mA for active VNS and control VNS, respectively). Only the research coordinator or assistant will set the device parameters based on randomized allocation. Therapists, assessors, and research participants will remain blinded to the assigned group throughout the study. In both groups, when a participant is actively attempting a task per protocol, the therapist will trigger the external stimulation device to pair active vs. sham VNS with the movement. Our study protocol provides detailed guidelines on the timing of pairing VNS during each task included in the protocol.

Participants in both groups will receive similar goal-directed and intense upper extremity rehabilitation. The therapy is focused on active movements, task specificity, high-number repetitions, variable practice, and active participant engagement. Tasks will be selected from 6 functional task categories: reach and grasp, gross movement, object flipping, simulated eating tasks, inserting objects, and opening containers. Progress will be ensured by adjusting the difficulty level and maintaining participant engagement. Approximately 30–50 repetitions will be performed in each category. On average, 300–500 repetitions will be performed within 120 min during each session.

After completing in-clinic therapy (18 sessions), participants will be transitioned to a home-based rehabilitation (90 days) therapy program. Each participant will be prescribed a daily exercise program (5 sessions/week) based on their functional level and goals. Before each session, they will swipe a magnet over the device and perform prescribed exercises for 30 min. Each group will receive stimulation according to their randomized group (0.8 mA vs. 0.0 mA). Magnet activation of the device will result in a 0.5-s burst of VNS every 10 s for 30 min, irrespective of the number of movements. The stimulation setting for every magnet swipe will be recorded in the VNS IPG. The research therapist will contact the participants by phone every other week to check on adherence and adjust the exercise program as needed. A research assistant will also call participants weekly, who will document their home exercises and any concomitant therapies taking place. The therapist, assessors, and participants will remain blinded during this period. Duration of therapy session in clinic and at home were chosen based on current human stroke studies.

### Outcome measures

For the first study objective, to determine the safety and feasibility of pairing VNS therapy with rehabilitation, we will report adverse events (AEs) in 3 categories: VNS surgery-related, VNS therapy-related, and VNS device related (*see the section* “*Expected risks*”). AEs will be labeled according to severity based on their impact on the patient. An AE will be termed *mild* if it does not have a major impact on the patient, *moderate* if it causes the patient some minor inconvenience, and *severe* if it causes a substantial disruption to the patient’s well-being. AEs will be categorized according to the likelihood that they are related to the study intervention. Specifically, they will be labeled *definitely unrelated*, *definitely related*, and *probably related* to the study intervention.

To determine feasibility, we will report treatment sessions attended by each participant and the number of dropouts in each group. The paired VNS intervention will be considered feasible if an adherence rate of at least 80% is achieved (≥15 of 18 sessions completed) and if the attrition (dropout) rate is no higher than 20% in each study group. We expect participants in both groups to participate in at least 50% of the assigned daily home exercises. We are also collecting data on patient satisfaction with the assigned intervention and adequacy of blinding procedures at the end of the study through a custom patient satisfaction questionnaire.

For the second study objective, to determine the potential efficacy of pairing VNS with rehabilitation, we will perform assessments of arm motor functions, activities of daily living, and quality of life. We will measure a change in Graded Redefined Assessment of Strength, Sensibility and Prehension (GRASSP) (41) performance from baseline to immediately after in-clinic therapy, 30-day and the 90-day timepoint. An increase of 4 points or greater in GRASSP score will be considered a clinically significant improvement (42). Secondary clinical outcome measures are the Toronto Rehab Institute Hand Function Test (TRI-HFT) (43), Capabilities of Upper Extremity Questionnaire (CUE-Q) (44), Spinal Cord Injury Independence Measure-III self-care subscore (45), and Spinal Cord Injury-Quality of Life (SCI-QoL) (46). All these measures are part of the National Institutes of Health common data elements for SCI. All measurements will be performed at baseline, at post-treatment (day 1), and at the 30-day and 90-day follow-ups. The core NINDS Spinal Cord Injury Common Data Elements—demographics, social status, general health history, history of the injury event, and neurological classification [International Standards for Neurological Classification of Spinal Cord Injury (ISNCSCI)]—will be completed within 30 days of collection of baseline data.

Other secondary outcome measures include assessing pain with the International SCI Pain Basic Data Subset (version 2) (47). Depression will be measured by use of the 8-item Patient Health Questionnaire (PHQ-8) (48, 49), with the item assessing for suicidality removed (49) (Table 1).

**Table 1:** Study timeline and procedures.

	Screening	Pre-implant baseline	Implant and randomization	Pre-therapy baseline	In-clinic therapy	Post-1 day	Post-30 day	Post-90 day
Eligibility criteria	X							
Informed consent	X							
Pre-surgical assessment	X	X		X		X	X	X
Prior and concomitant medications	X	X		X	X	X	X	X
Device implant			X					
Randomization			X					
VNS + rehabilitation					active VNS or control VNS	Home based active VNS or control VNS		
SCIM-III (SC)		X		X		X	X	X
GRASSP		X		X		X	X	X
TRI-HFT		X		X		X	X	X
CUE-Q		X		X		X	X	X
SCI-QoL		X		X		X	X	X
ISCIPBDS		X		X		X	X	X
PHQ-8		X		X		X	X	X
PSQ						X	X	X
Adverse events			X	X	X	X	X	X
Therapy compliance					X	X		

VNS, Vagus Nerve Stimulation; SCIMII-SC, Spinal Cord Injury Independence Measure III-Self-Care; GRASSP, Graded Redefined Assessment of Strength, Sensibility and Prehension; TRI-HFT, Toronto Rehabilitation Institute Hand Function Test; CUE-Q, Capabilities of Upper Extremity Questionnaire; SCI-QoL, Spinal Cord Injury Quality of Life; ISCIBDS, International SCI Pain Basic Data Subset (version 2); PHQ-8, Patient Health Questionnaire; PSQ, Patient satisfaction questionnaire.

### Patient and public involvement

Patients and/or the public were not involved in the design of this protocol. However, we will invite the participants of this study to provide input on study procedures, such as the feasibility of attending in-clinic sessions three times per week and 30-min home exercise program, the relevance of the tasks included in the protocol to their daily life, the burden of data collection, adequacy of blinding procedures and their experiences with the study participation. This information will be considered to refine this protocol further for a larger RCT.

### Sample size, data collection and analysis

We plan to enroll 8 participants and randomize them 1:1 to the active VNS or sham VNS group to study the safety and feasibility and to inform the design of a larger trial. No sample size calculations were performed to study the potential efficacy. We base this sample size on budgetary considerations during funding period.

Case report forms will be the primary method of obtaining data. Data will then be directly entered into the electronic database. Prior to the start of the study, the investigator will complete a Delegation of Authority Form showing the signatures and handwritten initials of all individuals authorized to perform study tasks, specifically those authorized to make or change entries on case report forms. The investigator or designee will provide completed case report forms for each participant. All required data are to be recorded in the electronic database promptly. The electronic data will be stored on a secured server and will be backed up daily to mitigate the risk for data loss. The investigators (RK and NY) will be solely responsible for the accuracy of the data.

Most of the planned analyses are descriptive owing to the pilot nature of the proposed study. Continuous variables will be summarized by mean, SD, or median quartiles as appropriate and categorical variables will be summarized via frequency and percentages. Outcome variables will be summarized separately for each randomization arm in graphs or tables, following the intention-to-treat principle.

## Ethics and dissemination

### Informed consent

Informed consent will be obtained from eligible participants by the PI and/or the research coordinator, and potential participants will be given sufficient time to decide to participate. The research coordinator will be involved in the consent process if there is a conflict of interest between potential research participants and the clinician PI. All procedures will be fully disclosed to the participants, and we will give them as much information as possible about the intervention and the data analysis. The participants will be given the opportunity to ask questions. They will be reassured that their participation in this study is voluntary and that they have the right to withdraw from the study at any time. Any new findings developed during this research that may affect the participants’ willingness to continue will be provided to them. The participants will be asked to sign a Research Subject Authorization Confidentiality and Privacy Rights form to comply with HIPAA regulations. This protocol and the associated informed consent documents have been approved by the Committee for the Protection of Human Subjects (CPHS) at McGovern Medical School in the UTHealth Science Center at Houston (#HSC-MS-22-0579).

### Confidentiality of patient data

All patient records will remain confidential. Data with Protected Health Information (PHI) will be de-identified and given a participant ID found on the Linking Log. The Linking Log is a separate file in a separate folder located on the PI’s desktop computer.

All research staff must have the Collaborative Institutional Training Initiative (CITI) training, which covers information privacy and security training to minimize the risk for privacy breaches. Efforts to maintain the confidentiality of all participants are per standards set forth by the CPHS at McGovern Medical School in the UTHealth Science Center at Houston.

### Expected risks

VNS surgery-related risks will include postsurgical complications including but not limited to dysphagia, hematoma, hoarseness of voice, vocal cord paralysis, facial paralysis, Horner syndrome, bradycardia, oedema, pain, and postsurgical infection (50, 51). We expect <1% surgery-related unresolved AEs at the 90-day follow-up on the basis of the results of the clinical trial in stroke. Risks related to almost any surgery include but are not limited to anesthesia-related adverse effects, pain, blood clots, infection, and bleeding.

VNS therapy-related risks include changes in blood pressure, heart rate, and respiratory rate, autonomic dysreflexia, worsening spasticity, and pain at the stimulation site. We will monitor blood pressure, heart rate, pain score, and respiratory rate before and after each therapy session and as needed if the patient reports any symptoms of discomfort, dizziness, etc. We will also record any incidence of autonomic dysreflexia, worsening spasticity symptoms, and development of any abnormal sensation. Other possible VNS-related symptoms include dyspepsia, dysphagia, hoarseness, hiccups, cough, laryngospasm, nausea, pain, paraesthesia, and pharyngitis.

VNS device-related events are rare (fewer than 1 in 100 cases). The lead or stimulator could move or protrude from the skin. Other risks include the risk that confidentiality could be inadvertently compromised.

### Adverse event reporting

The investigators and/or research coordinator will be responsible for collecting and reporting AEs during the trial. Participants will be recommended and encouraged to report any unanticipated problems or AEs throughout the course of the study (from the time of consent to the completion of the study) to the research coordinator by email or phone. The research coordinator will communicate all unanticipated problems and AEs to the PIs, who will record all reportable events with start dates occurring any time after informed consent is obtained until 7 days (for non-serious AEs) or 6 weeks (for serious AEs) after the completion of the intervention. Records of unanticipated problems and AEs will include the date, the details of the problem/event, and when and how the problem/event was reported to the investigators. The PI will report problems according to the UTHSC-Houston IRB policy.

#### Safety monitoring plan

Participants will receive routine postsurgical care and follow-up after implantation. We will collect data on VNS surgery-related events (as detailed in the “Expected risks” section) and coordinate care with the surgeon for additional follow-up. We will monitor the participants’ vital signs during each therapy session (pre-and post-session) and as needed. We will also have a medical monitor consisting of 2 physicians with expertise in SCI at UTHealth Science Center at Houston who will review all AEs and significant AEs to determine the safety of continued participation of each participant in the study.

This study will be stopped prior to its completion if (1) the intervention is associated with adverse effects that call into question the safety of the intervention; (2) we experience difficulty in study recruitment or retention; (3) any new information becomes available during the trial that necessitates stopping the trial; or (4) other situations occur that might warrant stopping the trial. (5) Intolerance to individual sessions will be assessed on a case-by-case basis by the study investigators to determine the safety of continued participation in the study.

In the event of an IPG device malfunction, participants will be offered an evaluation by a neurosurgeon for removal or replacement of the device. If the participant prefers the device to be removed or replaced, they will be evaluated by a neurosurgeon.

At the end of the study, although the intention is to allow participants to keep the device so that long-term treatment is possible, some participants may prefer that the device be removed. If a participant discontinues the study or does not want further treatment, the generator and the portion of the lead coiled in the chest wound should be removed. The device is expected to last at least 5 years. If participants wish to continue therapy, the device is expected to be commercially available at that time for replacement surgery, and if a device is needed sooner during the study, one will be provided.

#### Dissemination plan

We anticipate publishing the results of the trial in a peer-reviewed journal within 1 year of study completion.

## Discussion

To our knowledge, this is the first triple-blind, randomized, sham-controlled study to test the safety, feasibility, and potential efficacy of the currently available VNS device (Vivistim System; MicroTransponder, Inc.). The proposed study aims to harness the brain’s neuroplasticity to help SCI patients reach a maximum level of recovery with the least invasive method with significant potential for long-term use in a home setting. Our proposed intervention, if successful, will remove several critical barriers to enable a drastic shift in rehabilitation interventions for improving upper extremity function for people with SCI. First, electrodes are directly implanted over the nerve, which enables better control of stimulation parameters. Second, once implanted, the intervention is easy to use in the clinic, in outpatient rehabilitation centers, and at home with minimal directions. The ease of use will allow rapid clinical translation of this intervention. Last, the intervention has potential to improve many other complications of SCI, such as depression, pain, and inflammation (33, 34, 52).

We believe the proposed protocol not only will allow us to test safety and feasibility but also will help our team do a test run of this complex study, which will allow us to refine the protocol and logistics specifically for a SCI population for a future larger RCT.

Limitations of this protocol include the small sample size and lack of a third control arm without VNS implantation. A third arm would be necessary for future studies to rule out any short-term or long-term risks or benefits of implantation. However, because of budgetary constraints, we could not incorporate a third arm and a larger sample size at this time. For similar budgetary reasons, we are unable to collect any relevant imaging data to study any mechanistic changes in the brain or spinal cord after the intervention. Another limitation of this study is lack of digitized measurement of hand grip strength, pinch strength. Additionally, there are no VNS dosing studies performed in humans, and the parameters used in this study are based on VNS studies in humans with stroke, where the proposed parameters were found safe and tolerable. Future VNS dosing studies in SCI are needed.

## Trial status

The study is actively screening and enrolling participants. Currently, four participants have been enrolled and implanted with a VNS device.
